# Supplementary material for: Widow spiders alter web architecture and attractiveness in response to same-sex competition for prey and mates, and predation risk
Source: Commun Biol. 2023 Oct 11;6:1028. doi: 10.1038/s42003-023-05392-y (PMC10567780; doi:10.1038/s42003-023-05392-y)
Supplement: Supplementary file 2 — Description of Additional Supplementary Files [file 42003_2023_5392_MOESM2_ESM.pdf]

Description of Additional Supplementary Files

File name: Supplementary Data 1
Description: The source data behind the paper and it's graphs.

File name: Supplementary Data 2
Description: Code to analyze data underlying the paper.
